# Chemical Composition and Disruption of Quorum Sensing Signaling in Geographically Diverse United States Propolis

**DOI:** 10.1155/2015/472593

**Published:** 2015-04-15

**Authors:** Michael A. Savka, Lucas Dailey, Milena Popova, Ralitsa Mihaylova, Benjamin Merritt, Marissa Masek, Phuong Le, Sharifah Radziah Mat Nor, Muhammad Ahmad, André O. Hudson, Vassya Bankova

**Affiliations:** ^1^The Thomas H. Gosnell School of Life Sciences, College of Science, Rochester Institute of Technology, 85 Lomb Memorial Dr., A350 Gosnell Hall, Rochester, NY 14623, USA; ^2^Institute of Organic Chemistry with Centre of Phytochemistry, Bulgarian Academy of Sciences, Academic G. Bonchev Street, Building 9, 1113 Sofia, Bulgaria

## Abstract

Propolis or bee glue has been used for centuries for various purposes and is especially important in human health due to many of its biological and pharmacological properties. In this work we showed quorum sensing inhibitory (QSI) activity of ten geographically distinct propolis samples from the United States using the acyl-homoserine lactone- (AHL-) dependent *Chromobacterium violaceum* strain CV026. Based on GC-MS chemical profiling the propolis samples can be classified into several groups that are as follows: (1) rich in cinnamic acid derivatives, (2) rich in flavonoids, and (3) rich in triterpenes. An in-depth analysis of the propolis from North Carolina led to the isolation and identification of a triterpenic acid that was recently isolated from Hondurian propolis (Central America) and ethyl ether of *p*-coumaric alcohol not previously identified in bee propolis. QSI activity was also observed in the second group US propolis samples which contained the flavonoid pinocembrin in addition to other flavonoid compounds. The discovery of compounds that are involved in QSI activity has the potential to facilitate studies that may lead to the development of antivirulence therapies that can be complementary and/or alternative treatments against antibiotic resistant bacterial pathogens and/or emerging pathogens that have yet to be identified.

## 1. Introduction

Propolis is a chemically complex substance collected by honeybees from regional macroflora [1]. Bees use propolis to strengthen the hive, to block holes and cracks in the hive, and to protect the hive from invading insects and microorganisms. The chemical constituents of propolis are related to bud, bark, and wound exudates collected by bees from accessible flora [2, 3]. As a result, the chemical composition of propolis depends on the local flora from a specific geographical region. Propolis is composed of resin and balsam, wax, essential and aromatic oils, pollen, and other organic materials [4, 5]. Over 300 compounds have been identified in different propolis samples and it is accepted that the flavonoids, aromatic acids, diterpenic acids, and phenolic compounds act as bioactive principles in different chemical types of propolis [6–8]. Propolis has been used in human medicine since the 17th century due to its biological properties pertaining to antibacterial, anticavity, antitumor, antioxidant, antiviral, anti-inflammatory, and immunomodulatory effects, in addition to other beneficial properties [8, 9]. Propolis is increasingly recognized as a factor in social immunity traits of the honeybee and these traits are important for increasing adult longevity, decreasing brood mortality, and increasing hive productivity [10]. A constituent of some propolis types, pinocembrin, has recently been shown to regulate immune genes in the honey bee* Apis mellifera* [11]. Thus, propolis is a diverse and rich natural product source to search for novel therapeutic compounds.

In bacteria, the expression of certain bacterial genes, including the virulome or the whole set of genes required for virulence, frequently depends on the cell density of the population. In one system, this phenomenon, termed quorum sensing (QS), is mediated by specific molecules called* N*-acyl-homoserine lactone (AHL) QS signals, also known as autoinducers [12–14]. The specificity of AHLs is determined by the acyl side chain length, degree of its saturation, and the presence or lack of an oxo-, or hydroxy-group in C3 position [15, 16]. AHLs are characterized as long- or short-chain AHLs depending on whether their acyl moieties consist of >8 or ≤8 carbon atoms, respectively [16]. Examples of bacterial phenotypes controlled by QS include conjugal transfer of plasmids, biofilm formation, swarming motility, virulence factor expression, bioluminescence, pigment production, and other traits [12, 13]. The QS mechanism in bacteria enables the regulation of gene expression and coordinated functions beneficial only when carried out by a large number of bacterial cells, for example, high cell density.

QS is important in host-microbial interactions in humans, marine systems, and some phytopathogens. Many Gram-negative bacterial species that use AHL-based QS contain a complete AHL QS regulatory circuit. This includes the QS core proteins known as LuxI-type protein (the AHL synthase) and a LuxR-type protein (the response regulator or receptor) [12, 17]. The manipulation of the regulation of QS may provide alternative and novel therapeutic approaches against bacterial pathogens [18, 19]. Details on the biochemical and molecular mechanism underlying QS regulation and developing approaches to manipulate QS regulated behaviors in bacteria have recently been investigated [18, 20, 21].

One approach to mitigate treatment towards bacteria that are resistant to clinically relevant antibiotic is to develop new methods of antipathogenic treatments that act to attenuate the expression of disease progression (virulence), which may be less likely to impose a selection pressure for the development of bacterial resistance [19, 22]. A strategy to develop novel antipathogenic treatments is by blocking the cell-to-cell communication mediated by QS systems. Recent studies have identified many natural and synthetic compounds as QS inhibitors (QSI) [18, 22, 23]. The majority of the QSI compounds have been shown to inhibit QS signaling in screens using AHL-dependent biosensor strains. A preliminary screening for QS inhibitors revealed that propolis displayed a qualitative inhibitor activity based on a single LuxR-based AHL-dependent biosensor strain [24].

In our previous work, we investigated the effects of commercially prepared propolis tinctures to affect QS-regulated responses using five different AHL-signal-dependent reporter strains, each containing a different LuxR-homolog receptor [25]. We showed that propolis samples that differ in their region of origin, chemical profile, and absorption spectrum exhibit different QSI responses and that these responses depend on the AHL-dependent receptor protein.

Studies investigating the chemical constituents and phytotherapeutic compounds of propolis from hives in the United States are underrepresented in the literature [26, 27]. In this work we report antiquorum sensing activity in ten propolis samples harvested from geographically diverse regions in the United States. The sampling locations represent distinct botanical characteristics and include samples from the cold North, the wet Southeast, and the dry Southwest regions of the United States. Furthermore, we determine the chemical profiles of each propolis provenience by GC-MS analysis and characterized the propolis into three groups by principal component analysis. Lastly, we identified pinocembrin, a flavonoid from propolis which we show disrupting AHL-dependent QS in bacteria.

## 2. Materials and Methods

### 2.1. Chemicals

All common chemicals and solvents are analytical reagent grade from worldwide recognized brands. Bis(trimethylsilyl)-trifluoroacetamide (BSTFA) and Lobar prepacked column size A [LiChroprep Si 60 (40–63 *μ*m)] were purchased from Merck. Polyamide 6 was purchased from Fluka. Pinocembrin was isolated from Bulgarian propolis as described in our earlier work [28]. Purified standards of* N*-acyl-homoserine lactones (AHLs) QS signals were purchased from Cayman Chemical Co. (Ann Arbor, MI, USA). Abbreviations for* N*-acyl homoserine lactones (AHLs) include the following: C4-HSL,* N*-butanoyl-homoserine lactone and 3-oxo-C12-HSL,* N*-3-oxo-dodecanoyl homoserine lactone. Bacto agar was purchased from VWR International, Radnor, Pennsylvania. The antibiotic gentamycin and other chemicals were purchased from Sigma-Aldrich (St. Louis).

### 2.2. Propolis Samples

The sample identification, geographic origin, and coordinates of the raw United States propolis used in this study are provided (Table 1).

### 2.3. Propolis Sample Preparation for Quorum Sensing Bioassay

Frozen propolis samples were ground to a fine powder and extracted with 70% ethanol by shaking for 24 hours. The insoluble materials were removed by centrifugation at 12,000 g for 10 minutes at 4°C. The extracts were diluted to 5% based on dry weight for AHL-based QS biosensor investigations as previously described by our group [25]. A translucent zone around the cellulose discs indicate quorum sensing inhibition due to the reduction of the AHL-dependent violacein synthesis and this is in clear contrast to a transparent zone which indicates death via cell lysis in the whole-cell biosensor strain CV026 as previously described in our work [16, 25].

Raw Hungarian propolis (prepared in 70% EtOH extract and based on 5% dry weight) and Hungarian propolis tincture (30% commercial) as previously studied in our laboratory were used as internal propolis standards [25], and both possess antiquorum sensing activity against CV026 as well as four additional whole-cell biosensors that monitor AHL-regulated QS activity.

### 2.4. CV026 Biosensor Strain in Reverse Bioassay

The potential of United States propolis to inhibit QS was tested in a reverse bioassay using the* Chromobacterium violaceum* strain CV026. In* C. violaceum* strain CV026, the LuxR homolog, CviR, regulates the production of a purple pigment, violacein, with exogenous short-chain (C4 to C8) alkanoyl or 3-oxo-alkanoyl side chain AHL [16, 29]. Violacein production in CV026 in the presence of short-chain AHLs is inhibited by the presence of long-chain AHLs (C10 to C14) thus inhibiting violacein production in the presence of the stimulator AHL in reverse bioassays (C6-HSL and C4-HSL) [15, 24]. This phenomenon allows the use of CV026 in reverse bioassay to identify compounds that disrupt AHL-mediated QS signaling. In this bioassay the long-chain AHL, 3-oxo-C12-HSL, was used as a positive control and impregnated into disc (2 *μ*L of 1 mM stock) to inhibit violacein production in the presence of inducing concentrations of short-chain C4-HSL AHL as previously shown in our laboratory [16, 25]. The extinction coefficient of CV026 AHL produced violacein has been determined to be 0.05601 mL *μ*g^−1 ^cm^−1^ [30]. The antibiotic gentamycin (disc impregnated with 10 *μ*g was used to visualize biosensor death as a transparent zone of growth inhibition.

### 2.5. Inhibition of Bacterial QS by Raw Propolis Collected in the United States

To test the effect of propolis extracts on the AHL-dependent phenotype (pigment production) of biosensor CV026, cellulose discs of 6 mm in diameter were impregnated with ethanol extracts of the ten propolis samples from the United States, placed on the surface of a soft agar plate seeded with strain CV026 induced with AHL C4-HSL, and incubated at 28°C overnight. Eight microliters of propolis at a 5% preparation in 70% EtOH was applied to the cellulose discs. Short-chain signal C4-HSL (final concentration of 2.42 *μ*M) combined with CV026 was added to a soft agar plate as previously described by our group [25, 29]. We used Hungarian propolis, previously studied by our group, as internal standards in the bioassays, since this propolis sample has been characterized for the inhibition of bacterial QS responses using five different whole-cell biosensors that contained different LuxR AHL signal receptor proteins [25]. The zone of inhibition was observed and measured as a translucent zone directly adjacent to the cellulose disc [25].

### 2.6. Quantification of Violacein Pigmentation

A 20 mm disc of the CV026 seeded soft agar medium below the discs containing the propolis was harvested and the violacein pigmentation was extracted and measured spectrophotometrically at *A*_585 nm_ as described by Blosser and Gray [31]. All experiments were repeated at least three times.

### 2.7. Extraction and Sample Preparation for GC-MS Analysis

Silylation was performed according to [32]. In brief, propolis, grated after cooling, was extracted twice with 70% ethanol (1 : 10, w : v) at room temperature for 24 h. A part of the ethanol extract (5 mL) was evaporated to dryness. About 5 mg of the extract was mixed with 50 *μ*L of dry (water-free) pyridine and 75 *μ*L of bis(trimethylsilyl)-trifluoroacetamide (BSTFA) and heated at 80°C for 20 min. The silylated extracts were analysed by GC-MS.

### 2.8. GC-MS Analysis and Identification of Compounds

The GC-MS analysis was performed with a Hewlett-Packard gas chromatograph 5890 series II Plus linked to a Hewlett-Packard 5972 mass spectrometer system (single quadrupole) equipped with a 30 m long, 0.25 mm i.d., and 0.5 *μ*m film thickness HP5-MS capillary column (Hewlett-Packard). The temperature was programmed from 60 to 300°C at a rate of 5°C/min and a 10 min hold at 300°C. Helium was used as a carrier gas at a flow rate of 0.8 mL/min. The split ratio was 1 : 10, the injector temperature 280°C, the interface temperature 300°C, and the ionization voltage (EI) 70 eV, *m*/*z* range 50–700.

The identification of individual compounds by GC-MS was performed using computer searches on commercial libraries, comparison with spectra of authentic samples, and literature data. If no reference spectra were available, identification was performed based on the mass-spectral fragmentation; in such cases for some compounds only tentative structures were proposed. Some constituents remained unidentified because of the lack of relevant references and information (all of them constituted minor percentage of TIC).

### 2.9. NMR Experiments

One- and two-dimensional NMR spectra (^1^H-, ^13^C-, DEPT, HSQC, and HMBC) were taken on Bruker AV 600, in CDCl_3_.

### 2.10. Isolation of Individual Compounds

Individual compounds were isolated from the sample NC-7 (North Carolina). The total 70% ethanol extract was concentrated and extracted successively with petrol ether (3x) and ethyl acetate (3x). The ethyl acetate extract was evaporated to yield 8.04 g dry extract, which was subjected to vacuum liquid chromatography on polyamide 6 eluted with chloroform-methanol-ethyl methyl ketone (20 : 2 : 1 to 20 : 12 : 6). Sixteen fractions were obtained. Fraction 3 (2.99 g), eluted with chloroform-methanol-ethyl methyl ketone (20 : 4 : 2), was rechromatographed on a column with polyamide 6 using chloroform-ethyl acetate (1% to 100%) as a mobile phase and 19 fractions were obtained. Fraction 3 (187.4 mg) was subjected to column chromatography on silica gel (Lobar) and eluted with chloroform-ethyl acetate (1% to 100%), and 20 fractions were obtained. Fraction 5 yielded 22 mg of cinnamyl-*p*-coumarate [33] and 6.9 mg benzyl-*p*-coumarate [33]. Fraction 7 yielded 5,5 mg of (*E*)-4-(3′-ethoxyprop-1′-enyl) phenol (ethyl ether of* p*-coumaric alcohol) [34], and fraction 15 gave 16,6 mg of 3-oxo-6*β*-hydroxy-lup-20(29)-en-28-oic acid [33].

### 2.11. Statistical Analysis

Multivariate analysis of propolis chemical profiles was performed by PCA, using the GC/MS data for groups of identified compounds expressed as a percentage of the TIC, respectively. Statistica Version 8.0 was used for the analysis. For biological tests, data were statistically analyzed using SAS (version 9.1; SAS Institute, Inc., Cary, NC, USA). All experiments were repeated at least three times.

## 3. Results and Discussion

### 3.1. QS Inhibitory (QSI) Activity of US Propolis Samples

We assessed whether propolis collected from different regions of the United States can disrupt the AHL-dependent QS response in biosensor CV026. All ten proveniences were initially screened for QS inhibitory (QSI) activity using the CviR-based (LuxR-receptor) AHL-dependent biosensor strain in reverse bioassays. We then selected six proveniences based on initial zone of inhibition responses in the reverse bioassay with CV026 and availability of sample (Table 2).

**Figure 1:**
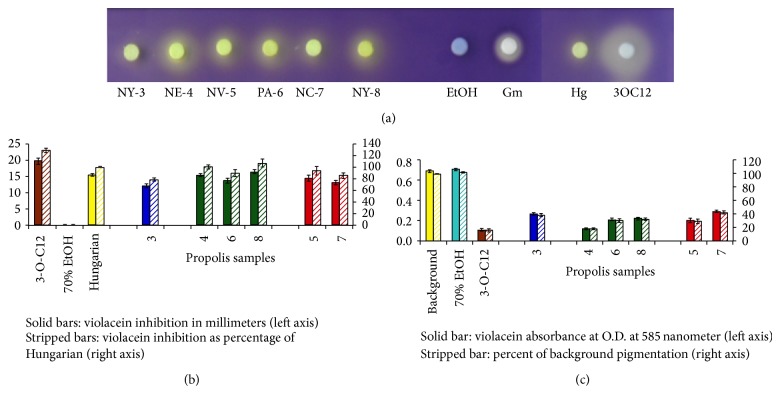
Inhibition of AHL-dependent violacein synthesis in* C. violaceum* strain CV026 in the presence of United States propolis. (a) Inhibition of AHL-regulated violacein synthesis in CV026 by the selected propolis in the disc diffusion assay. Abbreviations include the following: 3OC12, positive control disc impregnated with long-chain 3-oxo-C12-HSL AHL signal. Others include the following: Hg, an internal standard, the Hungarian raw propolis to visualize violacein synthesis inhibition as a translucent zone adjacent to the disc and as previously reported by our laboratory [25]; EtOH, pure solvent of 70% ethyl alcohol (control); and Gm, antibiotic gentamycin to visualize biosensor death as a transparent zone adjacent to the disc. All experiments were performed in triplicate and (a) are a representative result of one replication. (b) Quorum sensing inhibition (QSI) by propolis samples in millimeters indicated by a translucent zone of violacein synthesis across the cellulose disc. Data presented is mean ± standard deviation. Data is also presented as percent of the Hungarian raw propolis (internal standard) (Hg). (c) Quantification of violacein pigment synthesis inhibition in CV026 after treatment with propolis. This data is presented as percent of violacein present in the soft agar plate background (positive control, full induction of violacein synthesis). Using the propolis samples, no CV026 biosensor growth inhibition (only observed in the antibiotic gentimycin containing disc) was observed as identified by transparent zone around the cellulose disc. All experiments were repeated at least three times with representative data of one experiment shown.

**Figure 2:**
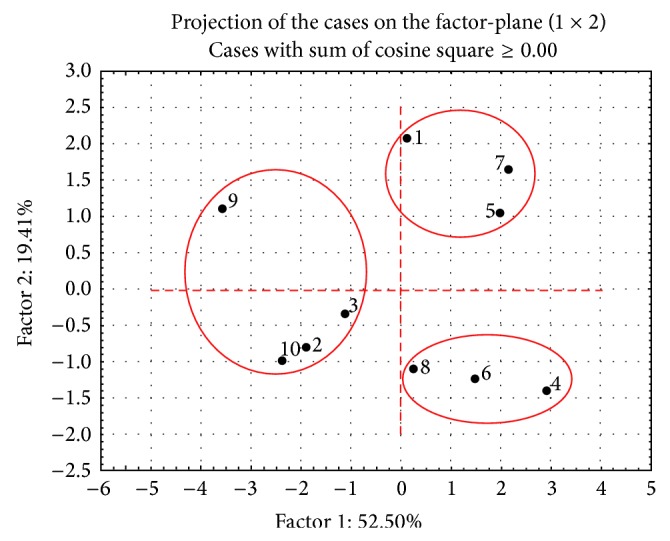
Principal component analysis (PCA) of the main classes of compounds identified. The numbers correspond to the sample numbers in Table 1.

The selected proveniences include the following: NY-3, NE-4, NV-5, PA-6, NC-7, and NY-8 (Figure 1). We quantified these six samples for their potential to disrupt AHL-dependent QS communication using the* C. violaceum* CV026 and propolis-containing cellulose discs: (1) to determine the size of the diffusion zone of inhibition (Figure 1(b)) and (2) to measure the amount of inhibition of the synthesis of the QS-regulated trait in CV026, violacein pigment production (Figure 1(c)). The differences between selected propolis samples are highly significant (*P* < 0.01). When compared to negative control (70% EtOH), all six samples had significantly larger zone of pigment inhibition and when compared to positive control (pure long-chain AHL, 3-O-C12), all treatments had significantly smaller zones of pigment inhibition. The NE-4, PA-6, and NY-8 propolis samples showed the largest zones of pigment inhibition adjacent to the propolis impregnated discs (Figure 1(a)), which were between 70 and 80% of the zone of inhibition observed with the pure long-chain 3-oxo-C12-HSL signal (positive control). Compared to the internal propolis standard, the Hungarian raw propolis, these three US samples were between 94 and 106% of the internal standard's pigment inhibition zone (Figure 1(b)).

The violacein was extracted from the soft agar samples of the zones of inhibition and quantified (Figure 1(b)). The amounts of pigment differed significantly (*P* < 0.01) for all propolis extracts when compared to the background pigmentation of randomly selected regions of the CV026-seeded soft agar distal to cellulose discs. When violacein amounts were compared to the background pigmentation, all extracts except the 70% EtOH (control) were significantly lower indicating disruption of violacein synthesis in the zone under and adjacent to the disc containing propolis. The six propolis samples ranged from 18 to 43% in comparison to the background pigmentation (100%) and 16% for the positive control pure long-chain 3-oxo-C12-HSL AHL signal (Figure 1(c)).

### 3.2. Chemical Profiles of US Propolis Samples

The chemical composition of all ten propolis samples from different regions of the United States (the States: New York, Nevada, Pennsylvania, North Carolina, and Louisiana) was analyzed by GC-MS after silylation. Over 60 individual compounds were identified. Distinct chemical profiles were observed for samples from different location, in accordance with the recent findings of Wilson et al. [10]. The chemical composition is presented by means of the main type of compounds identified (Table 3) and is obviously qualitatively and quantitatively variable. Detailed data about the percentage of the total ion current (TIC) in the mass chromatogram for individual constituents can be found in Table S1 (supplementary data) (see Table S1 in Supplementary Material available online at http://dx.doi.org/10.1155/2015/472593). In all samples analyzed, aromatic acids and their esters, flavonoids, and chalcones were found, while the presence of triterpenes was detected only in six samples. The triterpenes are compounds found in propolis from tropical and subtropical regions and have not been found in North American propolis samples.

Because of the complex chemical composition of the samples, the chemometric approach principal component analysis (PCA) was applied using the relative amounts of the main classes of compounds (percentage of TIC) from the GC-MS analysis. The obtained two-dimensional plot covers 72% of the total variation (Figure 2). Based on the PCA results, three groups of propolis samples were distinguished: one rich of cinnamic acid derivatives (group I, samples NY-2, NY-3, MN-9, and NY-10), a second group with high concentration of flavonoids (group II, samples NE-4, PA-6, and NY-8), and a third group characterized by relatively high concentration of triterpenes (group III, samples LA-1, NV-5, and NC-7). It is obvious that the samples from the first two groups originate predominately from Northern American states. Such differentiation is not surprising because in different climatic and geographical regions the bees collect resins from different plants.

The phenolic compounds identified in all samples (phenolic acids, esters, and flavonoids) are known propolis constituents found in poplar type propolis, the most widely distributed propolis type in the temperate regions [35–37]. This indicates that one of the propolis plant sources is representatives of genus* Populus*. In North America, poplars from section* Aigeiros*,* Tacamahaca,* and* Leuce* are widely distributed. The major constituents of group I propolis samples (NY-2, NY-3, MN-9, and NY-10) are aromatic acids and esters such as benzoic, cinnamic,* p*-coumaric, and ferulic acids and benzyl-*p*-coumarate. The high percent of aromatic acids and low concentration of flavonoids are characteristic for the bud exudates of* Populus* spp. of section* Leuce* [38], such as* Populus tremula* (aspen) in Europe and* Populus tremuloides* Michx. (American aspen) in North America.* P. tremuloides* is native to cooler areas of North America, regions where the samples NY-2, NY-3, MN-9, and NY-10 were collected. The American aspen is shown as a source of the Canadian propolis [32] and is the possible botanical source of the samples from Minnesota and New York. The sample from Minnesota is the only one containing significant amounts of phenolic glycerides. These compounds have been found in propolis from Russia and Switzerland [38, 39]. They are typical constituents of bud exudates of poplars belonging to the section* Leuce* [40]. Their mass spectra (of the silylated compounds) are highly characteristic and can be successfully used for positive identification [39, 40].

The propolis samples from group II (NE-4, PA-6, and NY-8) were characterized by poplar bud flavonoids such as pinocembrin, pinobanksin, pinobanksin-3-O-acetate, chrysin, galangin, and pinocembrin chalcone. The aromatic acids and esters were minor constituents. This chemical profile is typical for resins of poplar buds from section* Aigeiros* [41] and* Populus fremontii* is the most probable plant source. Recently, the American poplars from both the* Tacamahaca* (*P. trichocarpa* and* P. balsamifera*) and the* Aigeiros* sections (*P. fremontii*,* P. deltoides, *and* P. alba*) were identified as sources of propolis from some American states (Oregon and California, Minnesota) [27, 42]. This group (NE-4, PA-6, and NY-8) contained the samples that demonstrated the most pronounced effect on the QS, as seen in Figure 1. They displayed high concentrations of flavonoid aglycones (flavones, flavanones, and chalcones). This fact supports recent findings: flavonoids and especially flavanones have been shown to interfere with quorum sensing and reduce the expression of several QS-controlled genes [43].

Group III was characterized by significant amounts of triterpenes. Samples NV-5 and NC-7 in the third group are rich in flavonoids as well. However, the high amounts of triterpenes might interfere with the effect of the flavonoids on QS. Poplars from* Aigeiros* section are the main source of the propolis from the third group. Nevertheless, the considerable amounts of triterpenes found in these samples indicated participation of other source plants. Among the triterpenes, oleanolic and oleanonic acids were the major constituents in the samples from coastal regions, whereas in the sample from Nevada (NV-5) a series of lupane type triterpenes were detected.

The samples from Pennsylvania (PA-6) and New York (NY-8 and NY-10) also contained triterpenes but their amount was low (less than 1% of TIC) and there the “triterpene” source is obviously minor.

### 3.3. Isolation of Individual Compounds from Triterpene Containing Propolis

Among the samples analyzed, the one from North Carolina was a typical representative of a mixed type propolis with triterpenes as important compounds. This sample was chosen for further analysis in order to isolate and identify some of its bioactive constituents. From the ethyl acetate extract, after using vacuum liquid and column chromatography, four known natural compounds were isolated. They were identified by comparison of their spectra with literature data. By comparison of ^1^H-NMR and mass spectra with published spectra, cinnamyl-*p*-coumarate [32], benzyl-*p*-coumarate [32], and ethyl ether of* p*-coumaric alcohol [(*E*)-4-(3′-ethoxyprop-1′-enyl)phenol] [33] were identified. The triterpene 3-oxo-6*β*-hydroxy-lup-20(29)-en-28-oic acid was identified by analysis of its ^1^H-. ^13^C, DEPT, HMBC, and HSQC NMR spectra and comparison of ^1^H- and ^3^C-NMR spectra with literature data [44] as shown in Figure 4. The ethyl* p*-coumaryl ether is a new propolis constituent and the triterpenic acid has been isolated from Hondurian propolis (Central America) [32] as shown in Figure 4.

It is interesting to note that the chemical composition of propolis from group III is somewhat similar to propolis from Honduras. Lotti et al. [32] isolated a series of cinnamic acid derivatives from Hondurian propolis, as well as flavanones and triterpenes. The tree* Liquidambar styraciflua* L. (Honduras styrax) is suggested as a botanical source of these propolis constituents. Among the isolated compounds 3-oxo-6*β*-hydroxy-lup-20(29)-en-28-oic acid, benzyl* p*-coumarate, and cinnamyl cinnamate were identified as major compounds in Hondurian propolis. On the other hand, the balsam of* L. styraciflua* is characterized by predomination of cinnamyl cinnamate, and the above-mentioned triterpenic acid is one of the major triterpenes in the cones of* L. styraciflua*. All these constituents were detected in high relative concentrations in the samples from The United States LA and NC. As* L. styraciflua* is distributed in south and east regions of North America, it is possible that it plays a role as a secondary plant source of the samples analyzed. The sample from NV, although it contains triterpenes, has different triterpenic profile and lacks the cinnamyl derivatives typical for *Liquidambar*. Perhaps some other plants are the source of their triterpenes. In general, the search for the source plants in the USA requires further studies.

### 3.4. Quorum Sensing Inhibitory (QSI) Activity of Pinocembrin

**Figure 3:**
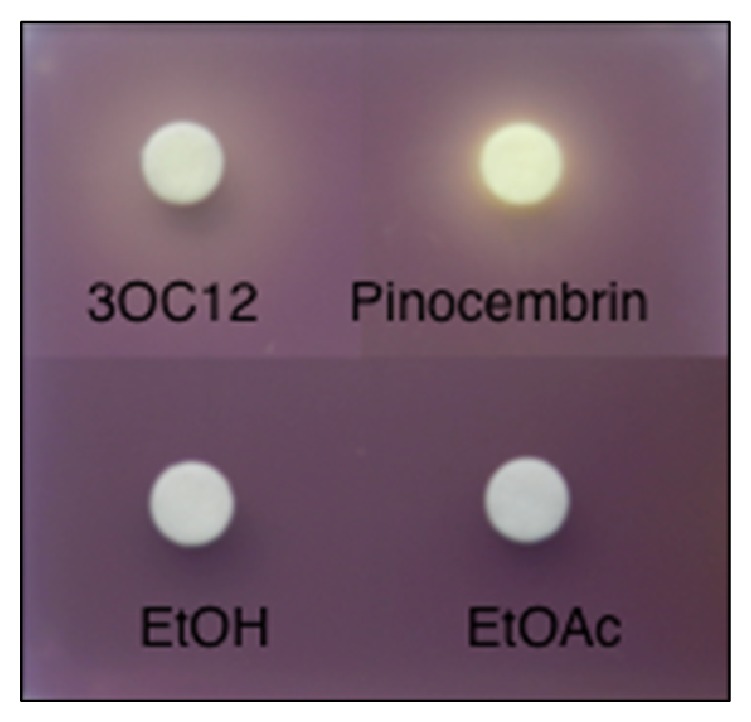
The flavonoid pinocembrin, which is present in the second group United States propolis, confers QSI activity. Abbreviations include the following: 3OC12, pure 3-oxo-C12 acyl-homoserine lactone (positive control); Pinocembrin, “European type propolis” fraction containing pinocembrin; EtOH, ethyl alcohol; EtOAc, ethyl acetate.

**Figure 4:**
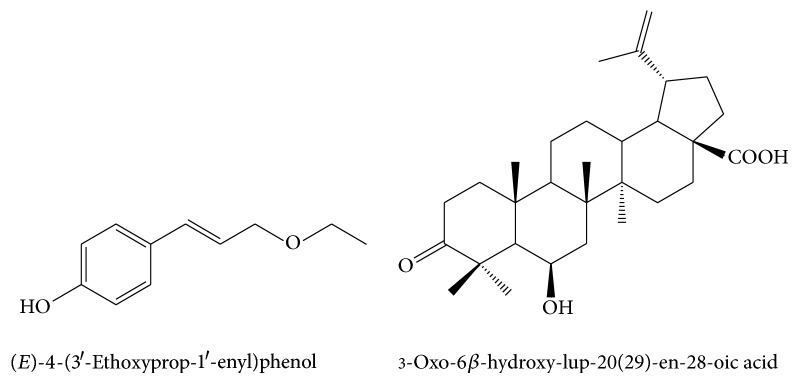


The United States group II propolis displayed QSI activity (Figure 1) and also contained poplar flavonoids including pinocembrin. Having previously purified a pinocembrin fraction from “European type” propolis, we tested the pinocembrin fraction for QSI activity in disc diffusion reverse bioassay using AHL-dependent biosensor CV026 as previously described. The pinocembrin fraction from the European-type poplar propolis displayed a translucent zone of inhibition around the disc, indicating inhibition of the AHL-dependent QS violacein synthesis consistent with pinocembrin exhibiting QSI activity (Figure 3). The translucent zone is similar in extent of response when compared to the positive control disc containing pure long-chain AHL 3-oxo-C12 homoserine lactone (3OC12). This is in contrast to the two solvents used, ethyl alcohol (EtOH) and ethyl acetate (EtOAc), that did not display QSI activity (Figure 3). Pinocembrin has been tested in mice and rats and appears not to be toxic up to levels of 40 and 100 mg kg^−1^ day^−1^ [45–47].

## 4. Conclusions

In this study, we analyzed 10 propolis samples from different geographic regions of the United States for QSI activity that disrupts QS AHL bacterial communication mechanism, correlated the QSI activity of propolis with its chemical composition, and identified the poplar flavonoid pinocembrin as a potential propolis active principle that disrupts AHL-dependent QS in bacteria. There are indications that additional flavonoids are important propolis constituents in this respect. It is obvious that propolis from the USA deserves further studies as a promising source of compounds and compound mixtures in the search for new approaches for antipathogenic treatments of bacterial pathogens based on natural products.

## Supplementary Material

Table S1. Chemical profile of US propolis samples obtained by GC–MS. Results are shown as % of total ion current.

## Figures and Tables

**Table 1:** United States propolis samples.

Sample ID	Origin	Geographic coordinates
LA-1	Baton Rouge, LA	30°22′41.4′′N 91°09′56.2′′W
NY-2	Beaver Dam, NY	41°25′50′′N 74°07′09′′W
NY-3	Dundee, NY	42°31′28′′N 76°58′29′′W
NE-4	Lincoln, NE	41°09′51.4′′N 96°28′57.9′′W
NV-5	Fallon, Nevada	39°28′22′′N 118°46′44′′W
PA-6	Millerton, PA	41°51′50′′N 76°57′18′′W
NC-7	Raleigh, NC	35°43′28.8′′N 78°40′33.4′′W
NY-8	Bloomfield, NY	42°53′57′′N 77°25′47′′W
MN-9	St. Paul, MN	44°59′27.8′′N 93°11′17.5′′W
NY-10	Yates Co., NY	42°39′36′′N 77°3′20′′W

**Table 2:** Quorum sensing inhibitory (QSI) activity of United States propolis samples.

Propolis origin, sample I.D.	QSI activity ZOI (mm)^a^	Used in additional QSI bioassays
Baton Rouge, LA (LA-1)	7	No
Beaver Dam, NY (NY-2)	7	No
Dundee, NY (NY-3)	10	Yes
Lincoln, NE (NE-4)	11	Yes
Fallon, NV (NV-5)	10	Yes
Millerton, PA (PA-6)	13	Yes
Raleigh, NC (NC-7)	14	Yes
Bloomfield, NY (NY-8)	14	Yes
St. Paul, MN (MN-9)	10	No
Yates Co., NY (NY-10)	7	No

*Internal standard* Hungarian (raw, prepared by our group and previously reported [24])	12	Yes
*Internal standard* Hungarian (tincture, commercial and reported in [24])	14	No

*Positive control:* long-chain AHL signal, 3-oxo-C12-HSL (1 uL of 1 mM)	17, QSI zone (translucent)^b^	Yes
*Antibiotic,* Gentamycin (2 uL of 50 mg/mL)	14, death zone (transparent)^b^	Yes

^a^ZOI: zone of violacein synthesis inhibition, as identified by a translucent halo adjacent to the 6 mm cellulose disc.

^b^A transparent zone of inhibition around the disc indicates death of the biosensor strain CV026 due to cell lysis whereas a translucent zone indicates inhibition of violacein synthesis in the AHL-dependent QS response in whole-cell biosensor CV026.

**Table 3:** Compound types identified in ethanolic extracts by GC-MS (% of TIC).

Compound type	LA-1	NY-2	NY-3	NE-4	NV-5	PA-6	NC-7	NY-8	MN-9	NY-10
Simple phenols and benzoic acid derivatives	1.9	15.7	12.4	0.4	0.2	2.5	0.5	1.9	13.1	16.6
Cinnamic acid derivatives	9.2	37.2	28.6	3.2	1.3	8.9	6.2	9.2	52.5	36.6
Fatty acids	0.3	1.1	0.3	0.2	0.2	0.7	0	1.8	0	1.7
Chalcones	5.2	6.8	4.5	18.2	8.2	7.2	9.9	6.0	2.5	3.4
Flavanones and dihydroflavonols	13.0	8.4	11.8	36.6	23.0	27.5	22.9	18.3	3.2	8.9
Flavones and flavonols	5.8	8.4	12.9	24.6	16.4	24.7	15.8	13.8	4.6	7.8
Phenolic glycerides	0	0.1	0.1	0	0	0	0	0	3.6	0.2
Triterpenes	20.9	0	0	0	6.9	0.6	11.5	0.4	0	0.7
Unknowns	0	0	0	0	8.0	0	9.2	1.4	0	0
