# Supplementary material for: Chemical Composition and Disruption of Quorum Sensing Signaling in Geographically Diverse United States Propolis
Source: Evid Based Complement Alternat Med. 2015 Apr 15;2015:472593. doi: 10.1155/2015/472593 (PMC4413979; doi:10.1155/2015/472593)
Supplement: Supplementary file 1 — Table S1. Chemical profile of US propolis samples obtained by GC–MS. Results are shown as % of total ion current. [file 472593.f1.doc]

**Table S1. Chemical profile of US propolis samples obtained by GC–MS. Results are shown as % of total ion current**

| **Compound** | **LA-1** | **NY-2** | **NY-3** | **NE-4** | **NE-5** | **PI-6** | **NC-7** | **NY-8** | **MN-9** | **NY-10** |
| --- | --- | --- | --- | --- | --- | --- | --- | --- | --- | --- |
| **Simple phenols and benzoic acid derivatives** | **1.9** | **15.7** | **12.4** | **0.4** | **0.2** | **2.5** | **0.5** | **1.9** | **13.1** | **16.6** |
| Benzylmethylketone | - | - | - | 0.2 | 0.1 | - | - | - | - | - |
| Benzyl alcohol | - | - | - | 0.2 | 0.1 | - | - | - | Tr* | Tr |
| Benzoic acid | 1.7 | 12.4 | 10.0 | Tr | - | 2.5 | 0.5 | 1.3 | 10.7 | 13.1 |
| hydroquinone | 0.2 | 0.1 | 0.3 | - | - | - |  | - | Tr | - |
| Vanillin | Tr | 2.3 | 1.7 | Tr | - | - | - | 0.4 | 1.6 | 2.4 |
| p-hydroxybenzoic acid | Tr | 0.5 | 0.4 | - | - | - |  | 0.2 | 0.8 | 0.8 |
| Vanillinic acid | Tr | 0.4 | Tr | - | - | - | - | - | - | 0.3 |
| Benzyl benzoate | - | - | - | - | - | - | - | - | 1.0 | - |
| **Cinnamic acid derivatives** | **9.2** | **37.2** | **28.6** | **3.2** | **1.3** | **8.9** | **6.2** | **9.2** | **52.5** | **36.6** |
| Cinnamic acid | 6.4 | 4.9 | 3.6 | - | - | 1.2 | 1.6 | 0.6 | 8.3 | 3.3 |
| *Z*-*p*-coumaric acid | 0.8 | 2.2 | 2.4 | - | - | - | - | 0.1 | 1.5 | 1.4 |
| *Z*-Ferulic acid | - | 0.6 | 0.8 | - | - | - | - | 0.6 | 0.7 | 0.8 |
| *E*-*p*-coumaric acid | 4.2 | 13.2 | 10.4 | 1.0 | 0.4 | 2.1 | 1.0 | 2.8 | 16.4 | 16.3 |
| *E*-Ferulic acid | Tr | 5.2 | 3.2 | 0.2 | 0.1 | 0.4 | - | 0.7 | 2.8 | 3.6 |
| Cinnanyl benzoate | - | - | - | - | - | 0.3 | - | - | - | - |
| Caffeic acid | Tr | 1.3 | 1.0 | 0.3 | 0.2 | 0.5 | 1.8 | 0.3 | 1.1 | 1.0 |
| Pentenyl coumarate | - | - | - | - | - | - | - | - | 0.5 | - |
| Pentenyl caffeate | - | - | - | - | 0.3 | - | - | - | - | - |
| Pentenyl caffeate | - | - | - | - | 0.3 | - | - | - | - | - |
| Benzyl coumarate Z | - | - | - | - | - | - | - | - | 1.0 | 0.6 |
| Cinnamyl cinnamate | 8.1 | - | - | - | - | - | 1.8 | - | - | - |
| Benzyl ferulate | Tr | Tr | 0.4 | - | - | - | - | 0.5 | 0.7 | 0.8 |
| Coumaryl benzoate | 1.6 | Tr | - | - | - | - | - | - | 0.9 | - |
| Benzyl coumarate | 1.3 | 4.0 | 2.9 | - | - | 1.0 | - | 1.2 | 6.0 | 4.2 |
| Coniferyl benzoate | - | - | - | - | - | - | - | - | 5.4 | 1.2 |
| Benzyl ferulate | Tr. | 3.1 | 2.0 | 0.4 | - | 0.8 | - | 1.4 | - | - |
| Benzyl ferulate | - | - | - | - | - | - | - | - | 1.7 | 2.3 |
| Benzyl caffeate | - | - | - | 0.7 | - | 0.5 | - | 0.7 | 1.0 | 0.7 |
| Cinnamyl *p*-coumarate | 5.3 | - | - | - | - | - | - | - | - | - |
| Coumaryl cimmanate | 3.3 | 1.5 | 1.0 | - | - | - | - | - | 1.8 | - |
| Coumaryl coumarate | - | - | - | - | - | - | - | - | 0.9 | - |
| Coniferyl cinnamate | - | - | - | - | - | - | - | - | 1.8 | - |
| Cinnamyl caffeate | 0.6 | 1.2 | 0.9 | 0.6 | - | 2.1 | - | 0.3 | - | 0.4 |
| **Fatty acids** | **0.3** | **1.1** | **0.3** | **0.2** | **0.2** | **0.7** | **-** | **1.8** | **-** | **1.7** |
| Stearic acid | 0.3 | 0.5 | 0.3 | 0.2 | 0.2 | 0.4 | - | 0.8 | - | 0.8 |
| Oleic acid | - | 0.6 | - | - | - | 0.3 | - | 1.0 | - | 0.9 |
| **Chalcones** | **5.2** | **6.8** | **4.5** | **18.2** | **8.2** | **7.2** | **9.9** | **6.0** | **2.5** | **3.4** |
| Pinostrobin chalcone | - | - | - | - | 0.6 | - | - | - | - | - |
| Pinocembrin chalcone | 3.8 | 5.9 | 4.5 | 17.6 | 7.6 | 6.8 | 7.2 | 5.3 | 2.5 | 3.4 |
| Tetraydroxychalcone | - | - | - | 0.3 | - | - | - | - | - | - |
| Trhydroxychalcone | 1.4 | 0.9 | - | 0.3 | - | 0.4 | 2.7 | 0.7 | - | - |
| **Flavanones and dihydroflavonols** | **13.0** | **8.4** | **11.8** | **31.6** | **23.0** | **27.5** | **22.9** | **18.3** | **3.2** | **8.9** |
| Pinocembrin | 2.0 | 2.8 | 3.5 | 10.9 | 5.7 | 5.9 | 5.3 | 4.5 | 1.6 | 3.4 |
| Pinobanksin | 3.7 | 1.3 | 4.8 | 5.2 | 1.4 | 6.3 | 1.5 | 5.2 | 0.8 | 2.7 |
| Sakuranetin | 4.8 | 2.0 | Tr | 5.9 | - | 7.5 | - | 3.3 | - | - |
| 3-acetyl pinobanksin | 2.5 | 2.3 | 3.5 | 9.0 | 6.3 | 7.5 | 5.8 | 4.0 | 0.8 | 2.8 |
| Pinobanksin 3-O-propanoate | - | - | - | - | 1.0 | - | - | - | - | - |
| Pinobanksin-3-O-butanoate | - | - | - | 0.3 | 1.5 | 0.3 | 1.5 | 0.5 | - | - |
| Pinobanksin-3-O-butanoate (iso) | - | - | - | - | 2.3 | - | 2.2 | - | - | - |
| Pinobanksin-3-O-pentanoate | - | - | - | 0.3 | 3.2 | - | 0.4 | - | - | - |
| Pinobanksin-3-O-pentanoate (iso) | - | - | - | - | 1.0 | - | 3.8 | 0.8 | - | - |
| Pinobanksin-3-O-pentenoate | - | - | - | - | 0.3 | - | - | - | - | - |
| Pinobanksin-3-O-hexanoate | - | - | - | - | 0.3 | - | 2.4 | - | - | - |
| **Flavones and Flavonols** | **5.8** | **8.4** | **12.9** | **24.6** | **16.4** | **24.7** | **15.8** | **13.8** | **4.6** | **7.8** |
| Galangin | 3.5 | 4.1 | 5.0 | 12.0 | 10.4 | 9.5 | 9.5 | 6.6 | 1.9 | 4.2 |
| Chrysin | 1.3 | 1.6 | 2.6 | 6.9 | 5.1 | 7.0 | 4.4 | 3.4 | 0.7 | 1.9 |
| Dihydroxymethoxyflavone | 1.0 | 0.8 | 0.7 | 1.9 | - | 2.4 | - | - | - | 0.6 |
| Kaempferol | - | 1.9 | 1.8 | 1.6 | - | 1.0 | 0.9 | 1.4 | 1.2 | 1.1 |
| Kaempferol Me ether | - | - | - | - | 0.9 | 1.6 | 0.4 | 0.4 | 0.8 | - |
| Quercetin methyl ether | - | - | 1.0 | 1.3 | - | 0.8 | - | 1.5 | - | - |
| Quercetin methyl ether isomer | - | - | 1.8 | - | - | - | - | - | - | - |
| Quercetin dimethyl ether | - | - | - | - | - | 0.9 | 0.6 | 0.5 | - | - |
| Quercetin methyl ether isomer | - | - | - | 0.5 | - | 1.5 | - | - | - | - |
| Quercetin methyl ether isomer | - | - | - | 0.4 | - | - | - | - | - | - |
| **Unknowns** | **-** | **-** | **-** | **-** | **8.0** | **-** | **9.2** | **1.4** | **-** | **-** |
| Unknoun (m/z 530, 342) | - | - | - | - | 2.0 | - | 2.1 | 0.6 | - | - |
| Unknoun (m/z 544, 342) | - | - | - | - | 1.8 | - | 1.3 | 0.8 | - | - |
| Unknoun (m/z 558, 342) | - | - | - | - | 1.0 | - | 0.6 | - | - | - |
| Unknoun (m/z 558, 342) | - | - | - | - | 1.2 | - | 1.5 | - | - | - |
| Unknoun (m/z 586, 342) | - | - | - | - | - | - | 1.7 | - | - | - |
| Unknoun (m/z 572, 342) | - | - | - | - | 2.0 | - | 2.0 | - | - | - |
| **Triterpenes** | **20.9** | **-** | **-** | **-** | **6.9** | **0.6** | **11.5** | **0.4** | **-** | **0.7** |
| Oleanolic acid | 3.7 | - | - | - | - | - | 3.6 | - | - | - |
| Ursolic acid | 0.8 | - | - | - | - | - | - | - | - | - |
| Oleanonic acid | 10.8 | - | - | - | - | - | 2.2 | - | - | - |
| Moronic acid | 4.0 | - | - | - | - | - | 0.7 | - | - | - |
| Ursonic acid | 1.6 | - | - | - | - | - | - | - | - | - |
| Triterpene (m/z 507, 421,375, 189) | - | - | - | - | 0.5 | - | 1.2 | 0.4 | - | - |
| Triterpene (m/z 507, 421, 375, 189) | - | - | - | - | 1.3 | - | 1.1 | - | - | - |
| Triterpene (m/z 507, 421, .,375, 189) | - | - | - | - | 0.6 | - | 0.3 | - | - | - |
| Triterpene (m/z 507, 421, 375, 189) | - | - | - | - | 0.4 | - | 1.7 | - | - | - |
| Triterpene (m/z 507, 421, 375, 189) | - | - | - | - | 1.2 | 0.6 | 0.3 | - | - | 0.7 |
| Triterpene (m/z 507, 421, 375, 189) | - | - | - | - | 2.9 | - | 0.4 | - | - | - |
| **Phenolic glycerides** | **-** | **0.1** | **0.1** | **-** | **-** | **-** | **-** | **-** | **3.6** | **0.2** |
| Acety-p-coumaroyl glycerol | - | 0.1 | 0.1 | - | - | - | - | - | 2.7 | 0.1 |
| Acetyl-di-p-coumaroyl glycerol | - | - | - | - | - | - | - | - | 0.9 | 0.1 |

*Tr - Traces
